# Remedial after-school support classes offered in rural Gambia (The SCORE trial): study protocol for a cluster randomized controlled trial

**DOI:** 10.1186/s13063-015-1081-7

**Published:** 2015-12-16

**Authors:** Peter Boone, Alpha Camara, Alex Eble, Diana Elbourne, Samory Fernandes, Chris Frost, Chitra Jayanty, Maitri Lenin, Ana Filipa Silva

**Affiliations:** Effective Intervention, London, UK; Brown University and Effective Intervention, Banjul, Gambia; London School of Hygiene and Tropical Medicine, Providence, USA

**Keywords:** Primary education, Development, Para-teachers, West Africa

## Abstract

**Background:**

Low education levels are endemic in much of the developing world, particularly in rural areas where traditional government-provided public services often have difficulty reaching beneficiaries. Providing trained para-teachers to teach regular after-school remedial education classes has been shown to improve literacy and numeracy in children of primary school age residing in such areas in India. This trial investigates whether such an intervention can also be effective in a West African setting with similarly low learning levels and difficult geographic access.

**Methods/Design:**

Design: cluster-randomized controlled trial.

Clusters: villages or groups of villages with 15–300 households and at least 15 eligible children in the Lower River and North Bank Regions of The Gambia.

Participants: children born between 1 September 2007 and 31 August 2009 planning to enter the first grade, for the first time, in the 2015–2016 school year in eligible villages. We anticipate enrolling approximately 150 clusters of villages with approximately 6000 children as participants.

Intervention: a program providing remedial after-school lessons, focusing on literacy and numeracy, 5 to 6 days a week for 3 years to eligible children, based on the intervention evaluated in the Support To Rural India’s Public Education System (STRIPES) trial (PLoS ONE 8(7):e65775).

Control: both the intervention and control groups will receive small bundles of useful materials during annual data collection as recompense for their time. If the education intervention is shown to be cost-effective at raising learning levels, it is expected that the control group villages will receive the intervention for several years after the trial results are available.

Outcomes: the primary outcome of the trial is a composite mathematics and language test score. Secondary outcomes include school attendance, enrollment, performance on nationally administered exams, parents’ spending on education, spillover learning to siblings and family members, and school-related time use of parents and children. Subgroup analyses of the primary outcome will also be carried out based on ethnic group, gender, distance from the main highway, parents’ education level, and school type.

The trial will run by independent research and implementation teams and supervised by a Trial Steering Committee.

**Discussion:**

Along with the overall impact of the intervention, we will conduct a cost-effectiveness analysis.

There are no major ethical issues for this study.

**Trial registration:**

Current controlled trials ISRCTN12500245. 1 May 2015.

## Background

### Motivation

It is widely recognized that one of the preconditions of a country’s successful development trajectory is a well-educated citizenry [2, 15, 17]. Across the developing world, educational attainment levels are often quite low and, even when students are attending schools, sometimes very little is learned [14]. This situation is more severe in rural parts of the developing world where monitoring public servants is harder [4] and, as our previous research has shown, the situation is particularly alarming in parts of rural West Africa [3].

Much recent work in education in the developing world has evaluated different methods of addressing these low learning levels and the difficulties in implementing public policy [6, 10]. The policy of training and providing para-teachers to teach supplementary classes after the normal school day has been shown to work in several settings in India [1, 7, 11]. The main purpose of this study is to implement such a program in rural Gambia and, using a cluster randomized controlled trial (RCT), assess its effectiveness in raising literacy and numeracy rates among primary-aged children there.

### Study setting

The trial will take place in The Republic of Gambia, located in West Africa. The Gambia has a population of approximately 1.9 million, of which 38.7 % are 14 years of age or younger [5]. It is characterized by substantial ethnic diversity. In addition to the official language (English), the tribal languages Mandinka, Wolof, and Fula are widely spoken.

In 2012, The Gambia’s Human Development Index value was 0.439, ranking it 165th out of 187 countries and territories measured [8]. Over 60 % of all Gambians depend on agriculture for their livelihood. Real per capita gross domestic product (GDP) is low, at around US$470 in 2013 [5]. Education levels are also low – despite a moderate gross primary enrollment ratio (82.0 % for boys and 84.0 % for girls), the net attendance ratio is quite low, at 40.0 % for boys and 45.0 % for girls [18]. Learning levels are also quite low. The United Nations Children’s Fund (UNICEF) Multiple Indicator Cluster Survey 2010 assessed literacy among women aged 15–24 at 61.2 % in urban areas and only 33.8 % in rural areas.

### The intervention

One method of dealing with low learning levels in rural areas is to identify individuals to provide supplementary after-school classes to reinforce lessons learned in school. Several of the authors of this protocol^1^^1^ partnered with the Naandi Foundation, an Indian non-governmental organization (NGO) which had been running such a program for several years, to implement the STRIPES RCT in rural Andhra Pradesh, India, to evaluate its effects on learning levels. We found that after 2 years, students in the para-teacher intervention arm of the trial scored 0.75 standard deviations (SD) higher on mathematics and language tests than the control group [7]. Similar interventions in other parts of India have also had large impacts on learning levels, catalogued in Muralidharan [11].

The intervention in this study will adapt the STRIPES para-teacher intervention to the Gambian context, providing remedial after-school education classes focusing on numeracy and literacy, 5 to 6 days a week, in each intervention village, to attempt to replicate the results from STRIPES [7] in a new context.

In intervention villages, we will deliver the following package of interventions:

- Supplementary remedial education classes
- Teaching and learning materials
- Student assessment and evaluation
- Community mobilization

*Supplementary remedial education classes*: the main component of the intervention is to provide additional educational support to children through a two and a half hour-long class, to be held five to six times a week before or after school hours and during vacations.

After-school classes (ASCs) will usually be conducted by residents of the community. They will be trained by the Effective Intervention technical team, in conjunction with appointed personnel from the Ministry of Basic and Secondary Education (MoBSE), to provide remedial lessons for the children of each target community in a space provided by the community. These “para-teachers” who will run the ASCs will be selected by the implementation team in consultation with the members of each intervention community. We will aim to hire only para-teachers who have completed the 12th grade, pass an Effective Intervention qualification exam, and reside or choose to reside in that particular village. In the absence of an individual with at least a 12th grade education, we will choose an individual who passes the qualification exam and offers the most suitable combination of proximity to the intervention village and level of schooling: e.g., someone with a 9th grade education who lives in a neighboring village.

A child-friendly pedagogy lesson plan, based on child participation, reflection, and interaction, with frequent use of teaching and learning materials (TLMs), will be prepared by the technical team based on the STRIPES intervention [7]. Para-teachers and monitors will be trained by Effective Intervention team members, in partnership with the Naandi Foundation, which developed and administered the STRIPES intervention, on the following subjects:

- Pedagogical best practices for ASCs
- How to facilitate English and mathematics learning during ASCs

The primary aim of an ASC is to ensure mastery of the concepts learned in regular classes during school hours.

The intervention will be provided to children from intervention villages planning to enroll in grade 1, for the first time, in the 2015–2016 school year. The intervention will consist of 3 years of ASCs, taking place in the child’s home village, spanning the 2015–16, 2016–17, and 2017–18 academic years, covering the first to the third grade curricula in consecutive years^2^. At the end of each academic year we will train the para-teachers to update their skill sets and to prepare them to teach the material in the next grade level. We will conduct capacity-building exercises for para-teachers and monitors throughout the trial using an in-service approach.

*Teaching and learning materials:* appropriate teaching/learning materials will be used in conjunction with oral instruction at the ASCs to help children understand concepts in English and mathematics. The materials will ensure children get time to practice and apply concepts taught to them in the classroom to increase the likelihood of mastery. These interactive materials are developed for language and mathematics corresponding to grade-specific competencies. They assist in reinforcing these competencies through repeated application and practice of each concept until mastery is reached. Intervention materials have been prepared based on the national curriculum and textbooks used in schools for each grade. The materials aim to explain, illustrate and practice the grade-level concepts covered in regular class. Table 1 describes the content of this bundle of materials.

**Table 1:** Materials for after-school classes (ASCs)

The Teacher’s Handbook	Worksheets
Provides a summary of each concept to be taught	Consist of exercise books given to children to practice and understand the concepts taught and learned through TLM and Material Cards
Introduces creative and interactive ways of teaching each concept	
Provides an overview of the competencies that need to be covered in each lesson of the curriculum	
The Teaching and Learning Material (TLM) Kit	Material Cards
Contains material to execute teaching/demonstration of a particular concept	Are used as self-learning tools
Provides a creative and interactive set of collaborative learning tasks for teachers and students	Have a variety of activities and exercises that initiate interaction among group members
Gives raw material with which to implement innovative pedagogical techniques such as story-telling through puppets, pictures, and charts for children	Provide multiple ways of making each child practice the concept learned and master the relevant competencies
Evaluation sheets	
Offer a set of questions and exercises based on the concepts taught Are administered periodically after covering a given number of concepts Are administered by the teacher to each child in the group	

### Assessment and evaluations

To objectively measure learning levels of children in order to inform the adaptive teaching strategy with the goal of reaching the desired learning level, the implementation team will conduct a monthly internal assessment test that will be prepared by the technical team and conducted by the para-teachers. These periodic assessments will be designed independently of the outcome assessments used for the primary outcome. They will be used to evaluate learning level progress and provide feedback used to improve the teaching and learning methods. There will also be a similar independent evaluation administered semi-annually or annually to only enrolled participants in intervention villages, for the intervention team to track its progress. The implementation team will share these assessments of individual and composite learning levels of children with parents and other community members with the goal of imparting a sense of shared responsibility for children’s learning outcomes.

### Community mobilization

Community sensitization, through mobilization campaigns and monthly meetings, will be conducted in the intervention villages to motivate children to attend academic support classes and to sensitize and engage parents and the greater community to the importance of education and their role in ensuring better outcomes. The process of community involvement is intended to galvanize families to take responsibility for their children’s attendance and performance in school and ASCs. From the start, communities will play a vital role in the program, as they are the ones responsible for identification of possible candidates for para-teachers. These candidates will be interviewed and tested by Effective Intervention. Communities will then participate in the final para-teacher selection.

The implementation team will work closely with the Village Development Committee (VDC) and School Management Committee (SMC) in each village, when available, to promote active participation and involvement of parents and communities in the process of children’s learning. VDCs and/or SMCs will be involved in the management of the academic classes and monitoring children’s attendance and their learning progress. Meetings will be conducted regularly with VDCs and SMCs.

### Objectives

Our main research question is whether the success of the STRIPES trial in providing an after-school para-teacher intervention to raise learning levels among primary school students in rural India can be replicated in rural parts of The Gambia. Our secondary research questions include whether this intervention changes families’ behavior vis à vis education, such as the care-giver’s and child’s time allocation, expenditure on schooling, and whether the intervention brings heterogeneous benefits to different subgroups of the population.

## Methods

### Study population and eligibility

This will be a cluster-randomized controlled trial conducted in eligible villages in the Lower River and North Bank Regions of The Gambia, which is located in West Africa. A village will potentially be eligible if the following conditions are met:

1. The number of households in the village is between 15 and 300 according to the 2013 Gambian census;
2. There are at least 15 children eligible for inclusion in the trial at the time of our enumeration^3^;
3. It falls inside an eligible cluster, determined by an enumeration and mapping exercise described below, designed to minimize the risk of bias from contamination;
4. The *Alkalo* (village chief) consents to allow the village to participate in the trial.

A child will be eligible if he or she is resident in a village within an eligible cluster, and fits the following criteria:

1. He or she did not attend first grade or higher in a lower basic school or madrasa in the 2014–2015 academic year;
2. The child’s caretaker intends to enroll the child in the first grade in the 2015–2016 academic year;
3. He or she is born between 1 September 2007 and 31 August 2009;
4. The caretaker consents to allow the child to participate in the trial.

Participants will be informed that they may withdraw from the trial at any time.

### Recruitment

We will perform a complete enumeration of children born between 2006 and 2010 in all villages meeting the criteria to be screened for eligibility in which the *Alkalo* consents. Participants will be recruited by the research team during enumeration.

### Randomization flowchart

**Fig. 1:**
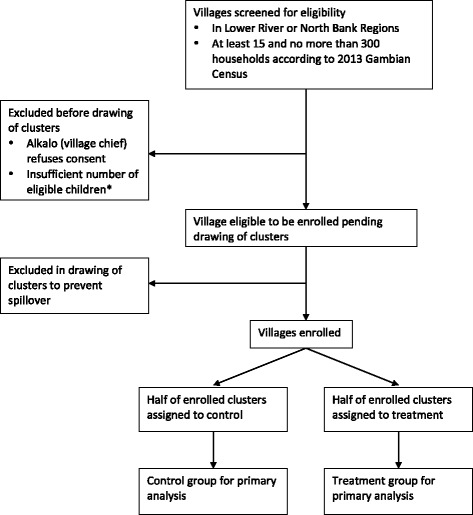
Flowchart of villages through the trial. Footnote: see eligibility criteria for details on criteria for eligibility of children and how the final minimum number of eligible children per village will be determined

A flowchart of how villages progress from screening for eligibility to inclusion in the final analysis is given in Fig. 1.

### Endpoints

The primary endpoint of this study is a composite of scores earned on English language and mathematics assessments from a test which will be conducted after 3 years of implementing the program: i.e. in spring 2018. The effect of the intervention will be expressed in SD units. Secondary outcomes to be analyzed will include:

- Performance on language and mathematics assessments, separately
- Performance on the third grade National Assessment Test, administered annually by the Gambian MoBSE to third grade students
- Subgroup-specific treatment effects to be investigated using interaction tests. Subgroups, defined at the individual level, to be considered are:
  - Ethnic group (Mandinka, Fula, Wollof, and other)
  - Gender
  - Geography (above or below the median distance from the main highway in each region)
  - Parents’ education level (in tertile groups)
  - School type (three types: public/government, private-religious, and private-non-religious)

- Spillover effects of the intervention on the reading ability of the child’s siblings immediately before and after the child in birth order and the child’s primary caretaker, as measured by a simple Annual Status of Education Report-style literacy test [13]
- Whether or not the school’s mothers’ club is active
- Effects of the intervention on educational spending by families, attendance of teachers and children in regular school, and parent and child’s time spent on education-related activities (doing homework, helping child with homework, study) outside of school hours
- Children’s enrollment in school

### Randomization

Randomization will be performed at the cluster level with stratification by region (Lower River or North Bank) and distance to the region’s main road (above/below median).

### Identifying clusters

We will create clusters from villages in the 2 regions through the following exercise:

1. All villages with between 15 and 300 households according to the 2013 Gambian Census will be considered for inclusion in the trial;
2. An enumeration exercise conducted by our survey enumerators will identify the number of eligible children in each village;
3. Those villages meeting the eligibility criterion for number of households and that have at least 15 eligible children^4^ will be considered for inclusion in eligible clusters;
4. Eligible clusters will be defined by using the mapping platform ArcGIS (https://www.arcgis.com) to map those villages satisfying eligibility criteria 1–3 and drawing clusters around groups of eligible villages using school catchment areas as a guide. In this stage, the clusters will be drawn such that no village in an intervention cluster is within a reasonable buffer zone (which we anticipate to be 3 km, but may vary depending on sample size requirements) of a village in a control cluster, excluding some villages from the enumeration, if necessary, to prevent risk of contamination.

### Control group

In the control group no after-school education program will be implemented during the period of the trial, but the children will receive small bundles of useful materials during annual data collection as recompense for their time. If the intervention is found to be effective in increasing child learning outcomes, and in the absence of insurmountable institutional or governmental barriers to doing so (e.g. the government rolls the intervention out across the country), control and intervention villages will receive the intervention for several years after the trial results are available.

### Duration

This program is scheduled to run the course of 3 full school years, starting in October 2015 and ending in June 2018 with the assessment.

### Participant timeline

January 2015–July 2015 → Enumeration – village consent, identifying eligible children, enrollment and obtaining parent consent for child participation

July 2015–August 2015 → Formation of clusters, including assessment of eligibility, randomization

September 2015-May 2018 → Participants receive intervention

June 2016, June 2017 → End of school-year data collection meetings in control and intervention villages

June 2018 → Assessment

### Sample size calculations

According to the regional directorates of the MoBSE in the Lower River and North Bank Regions [16], just under 10,000 grade 1 children are eligible to attend school in the combined North Bank and Lower River regions in the 179 public schools in these regions. The exact numbers of clusters and eligible students will not be known until after the first enumeration is carried out, but based on the above numbers we estimate that there will be around 150 clusters, each including an average of 40 students (6000 students in total). In the STRIPES trial the estimated effect was a 0.75 SD increase in mean score: however, effects of smaller magnitude than this would still be important to detect. Assuming that 60 % of the eligible children will take the test at the end of the trial and an intra-cluster correlation coefficient of 0.23 (as seen in the STRIPES trial), then a trial with 75 intervention villages and 75 control villages will give over 90 % power to detect a difference of 0.3 SD in the standardized score between intervention and control villages^5^ using a conventional 2-sided significance level of 5 %. If the treatment effect is of the order of that seen in the STRIPES trial then there will be reasonable statistical power to explore interactions by ethnicity, gender, wealth and geographic location.

### Assignment of interventions

Randomization of clusters will be performed by the trial statistician based in London using a random number generator. Randomization will be stratified by region (North Bank versus Lower River) and by above/below median distance to the main highway in each region. This is an unblinded study as, following randomization, participants will be aware of whether or not they are in an intervention or control village. Participants will be enrolled by the trial’s research arm, which will conduct all enrollment and data collection operations. This research arm will be operated entirely independently of the team delivering the intervention in the intervention group.

### Data collection, entry, and management

In all eligible villages we will collect the following information:

- Individual-level information about the parents/guardians of eligible children and about the children themselves at the beginning of the trial, child-level information at the end of the first and second academic years, and child, sibling, and parent/guardian-level information at the end of the trial
- The performance of eligible children on an EGRA/EGMA examination^6^, administered at the end of the trial, and on the National Third Grade Assessment Examination
- Village-level information on the access to various facilities and amenities in each eligible village
- School-level information about the school facilities and teachers, including attendance at school, observed during the course of the trial, of both students and teachers

Tests will be administered to all eligible children available in the village on the day of testing. In some cases, if a holiday, strike, or administrative necessity negatively impacts attendance at the test, a second visit to that village to reach all eligible and available children will be conducted.

The main challenge we foresee in maintaining the integrity of the data we collect is finding and motivating the children to take the test at the end of the trial period, particularly those children not receiving the education intervention. To address the motivation problem, we will provide all test takers and their caretakers with a small bundle of useful materials, such as snacks and writing utensils, as recompense for the children’s time in taking the test. To ensure we are able to locate as many enrolled control and intervention children as possible at the end of the trial for the test, we will have a “field day” in each village at the end of each academic year in which we will invite all of the eligible children in a village and their parents to a brief meeting in a central location in each village. This location will be chosen for its suitability as a place of test administration at the end of the trial. In the meeting, we will collect data on the children, including their enrollment in school, and on their parents. As recompense for the time spent at these meetings, we will provide attendees a small bundle of useful materials, similar to that mentioned above, for the final assessment. The goals of these meetings are to:

- Keep track of eligible children to ensure we are able to find as many as possible at the end of the third year
- Establish a place where testing can be done in each village in a fashion that is balanced between intervention and control arms, as not all villages have schools and intervention villages will establish such a place through the process of administering the intervention
- Establish and maintain familiarity of enrolled children with data collection efforts during the course of the trial to maximize turnout at the final assessment

Data will be collected on paper form by survey enumerators and entered into a database in Banjul, the capital of The Gambia. Data will be entered onto a server in Banjul and the database will be stored securely with ‘Cloud’ backup through a third-party service. All data will be double-entered and the database will provide an interface for resolving inconsistencies which will be adjudicated by an administrator consulting the paper forms and correcting the record accordingly.

### Statistical analysis

The primary analysis will follow the intention to treat principle, i.e. the participants will remain in the group they were randomized to and not analyzed according to the interventions actually received.

Child-specific composite test scores at the end of the third academic year will be compared between intervention and control groups using an analysis of covariance (ANCOVA) model. Stratification factors included in the randomization (and no others) will be adjusted for in the primary analysis. Provided normality assumptions hold, robust standard errors, allowing for the clustering, will be used here and elsewhere. Bootstrap confidence intervals will be reported if the normality assumptions are seriously violated. The adjusted difference in means will be divided by the SD of the test score in the control group to give a standardized difference, with a non-parametric bootstrap confidence interval (bias corrected and accelerated, 2000 replications) computed for this.

Secondary analyses will extend the ANCOVA model described above to (separately) investigate interactions by ethnic group, gender, wealth and geographic location. Sensitivity analyses will examine the effect on the treatment effect estimate of different treatment of missing data: e.g. imputation, assigning all missing values a zero, and other specifications according to Manski [9]. Program cost per 0.1 SD improvement in test scores will be calculated.

### Monitoring

As we anticipate no potential harms from this intervention, there will be no Data Monitoring Committee, interim analyses or stopping rules.

### Ethics and dissemination

Ethical approval for this study has been obtained from the London School of Hygiene and Tropical Medicine (LSHTM) Interventions Research Ethics Committee, approval dated 27 November 2014, ethics reference number 8767. Consent will also be obtained at the following levels:

- The national government level, from the MoBSE of The Gambia
- The village level, from each village’s *Alkalo*
- The household level, from the household head in each household where data are collected
- The caretaker level, from the caretaker of each eligible child

Any protocol modifications will be communicated to the LSHTM Ethics Committee and each consent level and consent will be re-obtained at the village and parent level at that point if deemed necessary by the LSHTM Ethics Committee.

All data will be kept strictly confidential – names will be removed from the database before analysis and the paper data collection instruments will be kept in a secure location in Banjul and destroyed after the statutory period expires.

We will disseminate the results of the trial to all enrolled villages and to the MoBSE through in-person meetings and delivery of study summary documents.

### Administrative structures

The trial will be run by independent research and implementation teams and supervised by a Trial Steering Committee. The trial will be jointly managed by the Field Research Manager, the Project Manager from the implementation team, and Effective Intervention senior staff.

### Implementation team

The team responsible for managing and implementing the education intervention will consist of:

- Para-teachers who will teach the remedial lessons to a group of 20 to 30 eligible children in each eligible village
- Monitors who will support the para-teachers’ work, monitoring their performance and evaluating children’s learning outcomes
- A Technical Team who will develop strategies, conduct trainings, and periodically supervise the work of para-teachers, monitors and children’s learning outcomes
- A Project Manager and several Project Officers who will coordinate and support all the work in the villages.

### Research Team

The team responsible for collecting the data that will evaluate the effectiveness of the project will consist of:

- Enumerators who will administer surveys to enroll children in the study and collect data from children’s families
- Supervisors who will lead field research teams in the field, supervising and facilitating the work of enumerators. They will also obtain consent from village *Alkalos* and school headmasters
- A Research Officer who will be responsible for monitoring teams (supervisors and fieldworkers) and facilitating and managing all data collection efforts
- A Field Research Coordinator who will lead the team and direct research activities
- Test Administrators
- Data Entry Operators who will enter the data received from the field
- A Data Entry Supervisor who will supervise data entry, printing of forms, and error management

## Discussion

Economic evaluation and sustainability of measures after intervention: along with the overall impact of the intervention, we will conduct a cost-effectiveness analysis in which we calculate the program cost for 0.1 SD improvement by the intervention.

Ethics/protection of human subjects: other than the consent processes discussed above, there are no major ethical issues for this study.

## Trial status

Clusters were randomized in September 2015 and the intervention is due to start January 2016.

## Abbreviations

ANCOVA: analysis of covariance
ASC: after-school classes
GDP: gross domestic product
LSHTM: London School of Hygiene and Tropical Medicine
MoBSE: Ministry of Basic and Secondary Education
NGO: non-governmental organization
RCT: randomized controlled trial
SD: standard deviation
STRIPES: Support To Rural India’s Public Education System
SMC: School Management Committee
TLM: teaching and learning materials
UNICEF: United Nations Children’s Fund
VDC: Village Development Committee.

## Footnotes

1 Boone, Eble, Elbourne, and Frost.

2 We chose to implement for 3 years instead of 2 (the length of the STRIPES intervention) to allow time for the “teething” process of learning how to implement the intervention in a new country, and because we are working in more marginalized areas than in the STRIPES project.

3 After we determine how many villages fit our initial eligibility criteria, the criterion specifying the minimum number of children may be relaxed downward to include enough villages to meet our sample size needs.

4 See previous footnote on the potential for downward revision of this minimum number.

5 This is a reasonable expectation in the context of similar interventions in the developing world [12] and is less than half of the improvement we measured in the trial on which this work is based [7].

6 These are assessments specifically designed to assess early grade reading and mathematics skills. More information on these instruments can be found at: https://www.eddataglobal.org/math/ and https://www.eddataglobal.org/reading/.
